# Scouring in Calves

**Published:** 1893-11

**Authors:** T. S. Gold

**Affiliations:** Commissioner on diseases of Domestic Animals. Board of Agriculture, State of Connecticut; West Cornwall, Conn.


### SCOURING IN CALVES.

Communication to JOURNAL by T. S. GOLD, Commissioner on diseases of Domestic Animals. Board of Agriculture, State of Connecticut.

---

The article by Dr. J. M. Parker, on "Scouring in Calves" (Oct. Number, page 227), in general meets my approval; but he has failed to notice two of the most striking forms of the disease.

First, young calves, often full blood Jerseys in good surroundings, are taken with scouring soon after birth and die in a few days. Some persons think it is one form, or result, or in some way connected with abortion, and no remedies seem to avail. They only suck their dams.

Second, calves that are suckled on cows for veal—new ones are bought as they are sold, and the business followed during the season. The stables become filthy and scouring sets in at any age, sometimes four to six weeks old, and calves more or less suddenly die, but may live several days or be sent to market after a moderate attack. This is evidently a contagious or infectious form, for the pens vacated for several months will communicate the disease to a new lot of calves. Both these forms resist treatment and are very serious.

The common dyspeptic diarrhœa yeilds to a cleaning up of the stable and medication. I usually for convenience of giving, prepare balls smaller than hens' eggs. Flour, enough to make adhesive Pulv. Ginger, and Prepared Chalk, same quanity. Moisten the whole with laudanum and essence of pepperment or Cholera mixture. Perhaps best to make balls one half size of hen's egg and give one or more twice a day. In cleansing the pens, fresh sods and loam are useful. Perhaps more thorough disinfection would relieve the contagious form, but the only help for the common farmer is to start with a new stock of calves in clean pens in the fields or distant barns.

WEST CORNWALL, CONN.,
Oct. 27th, 1893.

